# Sustained Tear Film Exposure After Parenteral Cefovecin Administration in Dogs: Pharmacokinetics and Relevance to Canine Bacterial Keratitis

**DOI:** 10.1111/vop.70238

**Published:** 2026-08-02

**Authors:** Meredith R. McClure, Lionel Sebbag, David J. Borts, Rachel A. Allbaugh, Jessica L. Payne, Melissa A. Kubai

**Affiliations:** ^1^ Department of Veterinary Clinical Sciences, College of Veterinary Medicine Iowa State University Ames Iowa USA; ^2^ Koret School of Veterinary Medicine Hebrew University of Jerusalem Jerusalem Israel; ^3^ Veterinary Diagnostic Laboratory, Department of Veterinary Diagnostic and Production Animal Medicine Iowa State University Ames Iowa USA

**Keywords:** bacterial keratitis, blood‐tear barrier, canine, pharmacokinetics‐pharmacodynamics, pharmacology, prolonged‐release formulation

## Abstract

**Objective:**

To characterize the tear film pharmacokinetics of parenterally administered cefovecin (Convenia) and compare tear concentrations with the minimum inhibitory concentrations (MICs) of common ocular pathogens.

**Animals Studied:**

Six healthy research‐bred Beagles.

**Procedures:**

Dogs received a single subcutaneous injection of cefovecin (8 mg/kg) after inducing blood‐tear barrier disruption through histamine‐mediated conjunctivitis. Tear fluid was collected using Schirmer strips at multiple time points up to 336 h and analyzed via liquid chromatography–mass spectrometry. Tear concentrations from both eyes were averaged for analysis. MIC values were determined for *Staphylococcus pseudintermedius* , *Streptococcus canis* , and *Pseudomonas aeruginosa* (*n* = 10 isolates per species).

**Results:**

In a pilot experiment (*n* = 1 dog), tear cefovecin concentrations were 1.5 to 430‐fold higher in the conjunctivitis eye than in the control eye at all but one time point. Across all dogs, tear concentrations ranged from 68 to 73 035 ng/mL and remained detectable throughout the 336‐h study. Mean ± SD pharmacokinetic parameters were: *C*_max_ 27.23 ± 27.74 μg/mL, *T*_max_ 61 ± 83 h, AUC 2734 ± 2563 μg/mL·h, and *t*_1/2_ 64 ± 46 h. Tear concentrations exceeded the MIC_90_ for *Streptococcus* (0.64 μg/mL) for 336 h and exceeded the MIC_90_ for some *Staphylococcus* (10.2 μg/mL) between 48 and 120 h, but remained below the MIC_90_ for *Pseudomonas*.

**Conclusions:**

Parenteral cefovecin achieved sustained tear concentrations sufficient to inhibit some susceptible *Streptococcus* and *Staphylococcus* isolates in vitro for clinically relevant time windows. While these findings support further investigation of cefovecin as an adjunctive therapy in selected cases, they do not justify empiric systemic antimicrobial use. Larger pharmacokinetic and clinical studies are warranted to validate these findings.

## 1 Introduction

Bacterial keratitis is a vision‐threatening condition that can result in severe pain, corneal perforation, and permanent visual impairment if not treated promptly and effectively. In dogs with moderate to severe bacterial keratitis, the standard of care relies on frequent topical antimicrobial administration, often at intervals of 1–2 h during the acute phase [1]. Although pharmacologically justified, such regimens pose substantial challenges to owner and patient compliance [2, 3]. Dosing frequency, difficulty of administration, patient temperament, and owner constraints all negatively influence adherence. In particular, treatment regimens exceeding four daily doses are consistently associated with a marked decline in adherence [4].

Beyond adherence, several pharmacokinetic and physiological factors inherently limit topical drug effectiveness at the ocular surface. Ophthalmic solutions have short precorneal residence times due to blinking, nasolacrimal drainage, and reflex tearing [5, 6, 7]. In dogs, tear film drug concentrations can decline rapidly; for example, topical prednisolone acetate concentrations decrease by approximately 45% within 1 min and by more than 95% within 15 min of administration [8]. These dynamics constrain the duration of meaningful antimicrobial exposure even with intensive topical dosing.

Due to the limitations of topical antimicrobial therapy, including poor patient tolerance of frequent dosing, owner compliance, and physiological barriers, long‐acting parenteral antimicrobials have been explored as potential adjunctive therapies. Tear film pharmacokinetics of systemically administered drugs have been described for a limited number of molecules across species, including doxycycline, minocycline, pradofloxacin, famciclovir metabolites, voriconazole, prednisone, melatonin, and cannabidiol [9, 10, 11, 12, 13, 14, 15, 16, 17, 18, 19]. However, sustained systemic exposure does not necessarily translate into therapeutically relevant tear concentrations; notably, extended‐release ceftiofur failed to achieve tear levels exceeding the MICs of common canine corneal pathogens [20]. Furthermore, systemic antimicrobial therapy may induce gastrointestinal adverse effects, including diarrhea and dysbiosis that can persist beyond the treatment period [21, 22, 23]. Therefore, antimicrobial stewardship principles emphasize that topical therapy remains the cornerstone of bacterial keratitis management, and antimicrobial selection should be guided by cytology, culture, and susceptibility testing whenever feasible [24]. Accordingly, any consideration of systemically administered antimicrobials for ocular surface disease must be restricted to carefully selected cases rather than used as an empiric alternative to topical therapy.

Cefovecin is a long‐acting, injectable, third‐generation cephalosporin approved for use in dogs. Following subcutaneous administration, cefovecin exhibits complete bioavailability, a prolonged apparent elimination half‐life, and high plasma protein binding (> 96%) [25]. Although high protein binding limits free plasma concentrations, the unbound fraction may be proportionally greater in extravascular compartments with lower albumin content, particularly under inflammatory conditions [25]. Cefovecin demonstrates in vitro activity against many clinically relevant *Staphylococcus* and *Streptococcus* species [26]; however, it is generally not considered a first‐line antimicrobial in most regions, and its off‐label use may be subject to local antimicrobial stewardship recommendations and prescribing regulations.

The objectives of this study were therefore: (i) to characterize cefovecin concentrations in the canine tear film following a single subcutaneous injection; (ii) to leverage a pharmacologic conjunctivitis model to approximate clinically relevant disruption of the blood‐tear barrier; and (iii) to determine MIC distributions for common canine ocular pathogens under albumin‐containing conditions and compare these values with tear exposure. Collectively, these aims were designed to assess the pharmacologic plausibility of highly selective adjunctive use, rather than routine treatment, of cefovecin in dogs with bacterial keratitis.

## 2 Materials and Methods

### 2.1 Animals

Six Beagle dogs from the Iowa State University research colony were enrolled (three castrated males, three spayed females; ~2.5 years old; 7.7–10.6 kg). All dogs were deemed healthy based on a complete physical and ophthalmic examination by a board‐certified ophthalmologist (M.A.K.), including CBC, serum chemistry, urinalysis, STT‐1, fluorescein staining, rebound tonometry, slit‐lamp biomicroscopy, and indirect ophthalmoscopy. Dogs were group‐housed and wore Elizabethan collars throughout the study. Daily physical examinations and vital sign assessments were performed.

### 2.2 Study Design

#### 2.2.1 Pilot Study

A pilot study assessed the impact of conjunctivitis on tear cefovecin concentrations. A one‐year‐old, castrated male Golden Retriever received topical histamine in one eye and artificial tears in the contralateral eye (control). A sterile 1% histamine solution was prepared by combining histamine dihydrochloride powder with 1.4% polyvinyl alcohol lubricating drops. This concentration was selected to induce moderate, self‐resolving conjunctivitis as a model of blood‐tear barrier disruption [27, 28, 29]. One drop was administered 20 min before cefovecin dosing and before each tear sampling time point. Cefovecin sodium (8 mg/kg) was administered subcutaneously. Tear samples were collected with Schirmer strips placed in the ventrolateral conjunctival fornix until wicking reached 20 mm. Sampling occurred at the following time points: 0, 0.25, 0.5, 0.75, 1, 1.5, 2, 4, 8, 12, 24, 36, 48, 72, 120, 144, 168, 192, 216, 240, 288, and 336 h post‐antibiotic administration. Plasma samples were collected at 0, 168, and 336 h. All samples were stored at −80°C.

#### 2.2.2 Experimental Study

Based on pilot findings, all six Beagles received 1% histamine ophthalmic solution in both eyes 20 min before cefovecin administration and before each tear sampling time point. Dogs received a single subcutaneous injection of cefovecin sodium (8 mg/kg). Tear and plasma collection mirrored the pilot approach and sampling schedule.

### 2.3 Cefovecin Quantitation in Tears

Cefovecin analytical standard (Zoetis) and cefpodoxime internal standard (Sigma) were prepared at 1 and 5 μg/mL, respectively, in a 50/50 acetonitrile/water mixture. The stock cefovecin solution was diluted to 100, 50, 10, and 1 μg/mL for the standard curve and quality control samples. Calibration standards and QC samples were prepared in pooled blank canine tears, which were collected from healthy dogs using polyvinyl acetyl sponges [30]. Prior to extraction, 18 μL of calibrant/QC was applied to blank Schirmer strips. Samples were extracted with 1 mL of methanol/acetonitrile/water (25:25:50) containing cefpodoxime, refrigerated, mixed, centrifuged, and the supernatant was transferred to autosampler vials. Quantitation was performed with LC–MS using a Vanquish Flex LC system coupled to an Exploris 120 Orbitrap mass spectrometer.

### 2.4 Minimum Inhibitory Concentrations

Ten clinical isolates each of *Staphylococcus pseudintermedius* , *Streptococcus canis* , and *Pseudomonas aeruginosa* (*n* = 30 total) obtained from canine corneal ulcers were evaluated. Microbroth dilution was performed in albumin‐containing conditions to approximate tear film protein environments [31]. Cefovecin was tested across serial concentrations (final well range 160–20 480 ng/mL after dilution adjustments), and plates were incubated per CLSI recommendations for each organism. Growth was assessed using a digital MIC viewing system.

### 2.5 Data Analysis

MIC_50_ and MIC_90_ were defined as the lowest cefovecin concentrations inhibiting growth in 50% and 90% of isolates, respectively. Paired *t*‐tests compared tear cefovecin concentrations between control and conjunctivitis eyes in the pilot study. Right versus left eye tear concentrations were compared at each time point in the experimental study; since differences were not detected (*p* ≥ 0.10), bilateral concentrations were averaged for analysis. Noncompartmental PK analysis used PKanalix (2019R1) with the linear‐log trapezoidal rule for AUC estimation. Statistical significance was set at *p* < 0.05.

## 3 Results

**FIGURE 1:**
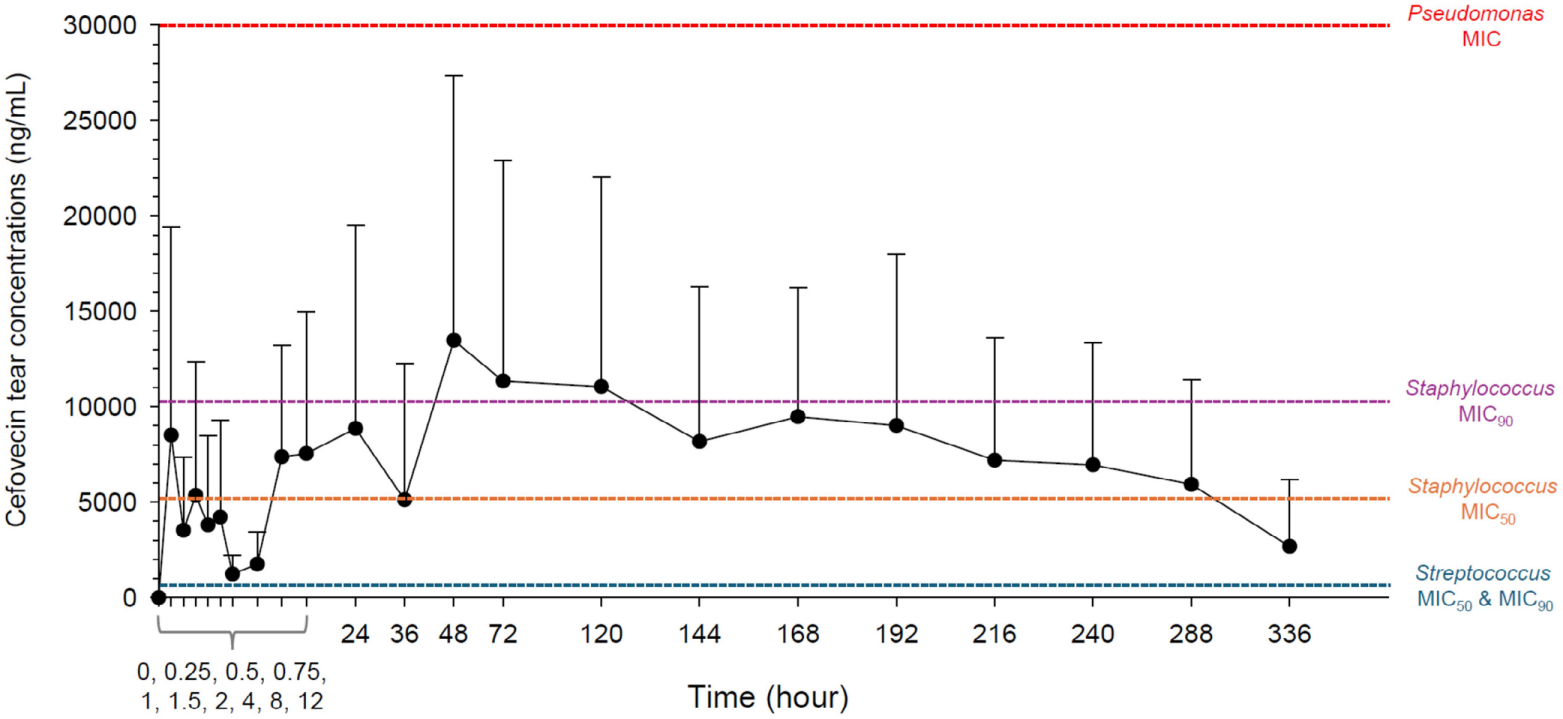
Mean + SD tear film concentrations of cefovecin over time following a single subcutaneous injection (8 mg/kg) in six dogs. Horizontal dashed lines denote MIC_50_ and MIC_90_ values for *Streptococcus canis* , *Staphylococcus pseudintermedius* , and *Pseudomonas aeruginosa* , determined under albumin‐supplemented conditions. Time points within the first 12 h are displayed at expanded resolution to improve visualization of early tear concentration changes. MIC, Minimal inhibitory concentration.

In the pilot experiment, cefovecin was detected in tears at all sampling times except baseline (*t* = 0). Tear cefovecin concentrations ranged from 0 to 183 ng/mL in the control eye and from 0 to 14 190 ng/mL in the conjunctivitis eye (*p* < 0.001; Table 1), corresponding to 1.5‐ to 430‐fold differences across time points. Plasma cefovecin concentrations ranged from 33.5 to 51.8 μg/mL. Across the six Beagles, cefovecin was detectable in tear samples at all time points through 336 h. Individual dog tear concentrations ranged from 68 to 73 034 ng/mL with mean tear concentrations at each time point depicted in Figure 1. Mean ± SD PK parameters were: *C*_max_ 27.2 ± 27.7 μg/mL; *T*_max_ 61 ± 83 h; AUC 2734 ± 2563 μg/mL·h; and *t*_1/2_ 64 ± 46 h. Average plasma concentrations ranged from 3.6 to 24.8 μg/mL, with a measured *C*_max_ of 23.0 μg/mL.

**TABLE 1:** Tear film cefovecin concentrations in a pilot experiment involving a single dog with histamine‐induced conjunctivitis in one eye and a contralateral healthy control eye following a single subcutaneous injection (8 mg/kg).

Time (hours)	Cefovecin tear concentrations (ng/mL)		
	Control eye	Conjunctivitis eye	Fold difference
0	0	0	0
0.25	3.7	5.7	1.5
0.5	5.3	680	128.3
0.75	37	242	6.5
1	52	140	2.7
1.5	135	1081	8.0
2	110	221	2.0
4	38	3663	96.4
8	80	3581	44.8
12	68	819	12
24	90	429	4.8
36	33	14 190	430
48	183	82	0.4
72	33	628	19.0
120	28	93	3.3
144	21	69	3.3
168	30	120	4.0
192	17	56	3.3
216	25	124	5.0
240	17	53	3.1
288	23	75	3.3
336	12	45	3.8

*Note:* Tear samples were collected at serial time points up to 336 h. Fold difference represents the ratio of cefovecin concentration in the conjunctivitis eye to that in the control eye at each time point.

MICs for *Streptococcus canis* ranged from 0.32 to 0.64 μg/mL (MIC_50_ = MIC_90_ = 0.64 μg/mL). Mean tear cefovecin concentrations remained above 0.64 μg/mL for the full 336‐h study period. MICs for *Staphylococcus pseudintermedius* ranged from 5.12 to > 20.5 μg/mL (MIC_50_ = 5.12 μg/mL; MIC_90_ = 10.24 μg/mL). Mean tear concentrations exceeded the MIC_50_ between 8 and 24 h and again between 48 and 288 h, and exceeded the MIC_90_ between 48 and 120 h. All *Pseudomonas aeruginosa* isolates grew at all tested cefovecin concentrations (MICs > 20.48 μg/mL), and mean tear concentrations remained below this level at all time points.

Percentage of time that free cefovecin concentrations remained above MIC (fT > MIC) was calculated. For *S. canis* , fT > MIC was 100%. For *S. pseudintermedius* , fT > MIC_50_ was 76.2%, whereas fT > MIC_90_ was 21.4%. For *P. aeruginosa* , fT > MIC was 0%.

## 4 Discussion

This study demonstrates that a single subcutaneous dose of cefovecin (8 mg/kg) produces sustained, quantifiable tear film concentrations for at least 336 h under conditions of histamine‐induced conjunctivitis. Tear exposure exceeded the MIC_90_ for *Streptococcus canis* throughout the entire sampling period and exceeded the MIC_90_ for *Staphylococcus pseudintermedius* for a more limited window between 48 and 120 h, while remaining consistently below the MIC thresholds for *Pseudomonas aeruginosa* . These findings contrast with prior work on extended‐release ceftiofur, in which tear concentrations failed to reach MIC targets for any common canine ocular pathogens [20].

For β‐lactam antibiotics, pharmacodynamic efficacy is best predicted by the fraction of time that free drug concentrations exceed the MIC (fT > MIC) [32]. In the present study, mean tear concentrations achieved 100% fT > MIC at the MIC_90_ threshold for *S. canis* , supporting sustained pharmacologic coverage for this organism. In contrast, for *S. pseudintermedius* , mean tear concentrations exceeded the MIC_50_ for much of the study period but exceeded the MIC_90_ for only 21.4% of the dosing interval. Because MIC_90_ values better reflect the upper range of susceptibility distributions and provide greater confidence for empiric coverage, the limited fT > MIC_90_ observed for *S. pseudintermedius* tempers expectations for consistent clinical efficacy against this organism.

The present findings must be interpreted explicitly within the framework of antimicrobial stewardship. Demonstration of sustained tear film exposure does not justify routine or empiric use of cefovecin for canine corneal ulceration. On the contrary, indiscriminate administration of long‐acting, critically important antimicrobials risks promoting antimicrobial resistance and undermines responsible ophthalmic care. Any consideration of parenteral cefovecin in the context of bacterial keratitis should be restricted to carefully selected cases, informed by cytology, culture, and susceptibility testing, and only when the expected pathogen spectrum aligns with cefovecin activity [24]. The long duration of action of cefovecin further heightens stewardship concerns, as inappropriate selection cannot be readily reversed once the drug is administered.

Marked interindividual variability in tear cefovecin concentrations further limits the strength of inferences drawn from mean concentration‐time profiles alone. Even when mean tear concentrations exceeded MIC thresholds, minimum concentrations frequently fell below those targets, indicating that not all dogs achieved equivalent ocular surface exposure. In addition, the tear concentration‐time profile was nonlinear, characterized by an early decline followed by delayed peak concentrations and gradual elimination. Similar nonlinear tear pharmacokinetics following systemic drug administration have been reported in other species and may reflect a time‐dependent distribution from plasma into the tear compartment via conjunctival vasculature and/or lacrimal gland contribution [9, 33].

The pronounced effect of conjunctivitis on tear cefovecin concentrations observed in the pilot study highlights the importance of blood‐tear barrier integrity. Breakdown of this barrier permits leakage of albumin‐rich plasma proteins into the tear film, facilitating transport of highly protein‐bound drugs such as cefovecin to the ocular surface [25, 27, 31]. The use of albumin‐supplemented media for MIC testing represents a methodological strength of this study, as standard susceptibility assays may underestimate the impact of protein binding on antimicrobial activity. Albumin concentrations used here approximate those reported in inflamed canine tears, thereby improving the physiologic relevance of MIC estimates [31].

Several limitations should be acknowledged. The study population consisted of young, healthy dogs of a single breed, limiting extrapolation to older dogs, brachycephalic breeds, and animals with naturally occurring keratitis or systemic disease. Conjunctivitis was pharmacologically maintained throughout the study period; in clinical settings, resolution of inflammation would be expected over time, potentially reducing tear penetration of cefovecin as the blood–tear barrier recovers. Additionally, while Schirmer strip sampling is widely used for tear pharmacokinetic studies, reflex tearing may dilute drug concentrations, although prior work suggests that tear flow rate may have a limited impact on measured concentrations for systemically administered drugs [12].

In summary, parenteral cefovecin may provide prolonged tear exposure that could be beneficial as an adjunctive therapy for susceptible *Streptococcus canis* and some susceptible *Staphylococcus* isolates, in circumstances where frequent topical administration is not feasible. However, cefovecin should not be expected to replace topical antimicrobial therapy, nor to provide reliable coverage against *Pseudomonas aeruginosa* . For *Staphylococcus pseudintermedius* , the observed exposure window may be meaningful for a subset of dogs and isolates, but inter‐individual variability and limited fT > MIC_90_ emphasize the need for caution and reinforce that cefovecin should not be used empirically or indiscriminately.

Future studies should evaluate cefovecin tear pharmacokinetics in dogs with naturally occurring bacterial keratitis, incorporate dynamic inflammatory trajectories, and integrate PK/PD modeling to estimate the probability of target attainment across MIC distributions. Such work will be essential to define if and in which narrowly defined scenarios parenteral cefovecin can be responsibly incorporated into multimodal treatment strategies while preserving antimicrobial stewardship.

## Author Contributions

**Jessica L. Payne:** investigation. **David J. Borts:** writing – review and editing, formal analysis, methodology. **Meredith R. McClure:** methodology, investigation, writing – original draft, visualization. **Lionel Sebbag:** formal analysis, conceptualization, methodology, writing – review and editing, visualization. **Melissa A. Kubai:** conceptualization, investigation, writing – review and editing, methodology, resources. **Rachel A. Allbaugh:** writing – review and editing.

## Funding

Funding was provided by an Iowa State University Veterinary Clinical Sciences (ISU VCS) Incentive Grant (SG2705634 assignee Dr. Kubai) and ISU VCS Start‐up Funds (Dr. Kubai).

## Disclosure

The authors have not used AI to generate any part of the manuscript.

## Ethics Statement

This study complies with the ARVO Statement for the Use of Animals in Ophthalmic and Vision Research and was approved by the Institutional Animal Care and Use Committee of Iowa State University (IACUC 22‐049).

## Conflicts of Interest

The authors declare no conflicts of interest.

## Data Availability

The data that support the findings of this study are available from the corresponding author upon reasonable request.
